# Structural, genomic information and computational analysis of emerging coronavirus (SARS-CoV-2)

**DOI:** 10.1186/s42269-022-00861-6

**Published:** 2022-06-15

**Authors:** Deepak Agarwal, Imran Zafar, Syed Umair Ahmad, Sujit Kumar, Qurat ul Ain, Jitendra Kumar Sundaray, Mohd Ashraf Rather

**Affiliations:** 1 Tamil Nadu Dr. Jayalalithaa Fisheries University-IFPGS, OMR Campus, Vaniyanchavadi, Chennai, India; 2 Department of Bioinformatics and Computational Biology, Virtual University Punjab, Lahore, Pakistan; 3 Department of Bioinformatics, Hazara University Mansehra, Mansehra, Pakistan; 4 Postgraduate Institute of Fisheries Education and Research Kamdhenu University, Gandhinagar, India; 5 Government College Women University, Faisalabad, Pakistan; 6 ICAR-Central Institute of Freshwater Aquaculture, Kausalyaganga, Bhubaneswar, Odisha India 751002; 7 Division of Fish Genetics and Biotechnology, Faculty of Fisheries Ganderbal, Sher-e- Kashmir University of Agricultural Science and Technology, Kashmir, India

**Keywords:** SARS-CoV-2, Genomic information, Structural biology, Drugs, Prevention

## Abstract

**Background:**

The emerging viral pandemic worldwide is associated with a novel coronavirus, SARS-CoV-2 (severe acute respiratory syndrome coronavirus 2). This virus is said to emerge from its epidemic center in Wuhan, China, in 2019. Coronaviruses (CoVs) are single-stranded, giant, enveloped RNA viruses that come under the family of coronaviridae and order Nidovirales which are the crucial pathogens for humans and other vertebrates.

**Main body:**

Coronaviruses are divided into several subfamilies and genera based on the genomic structure and phylogenetic relationship. The name corona is raised due to the presence of spike protein on the envelope of the virus. The structural and genomic study revealed that the total genome size of SARS-CoV-2 is from 29.8 kb to 29.9 kb. The spike protein (S) is a glycoprotein that attaches to the receptor of host cells for entry into the host cell, followed by the attachment of virus RNA to the host ribosome for translation. The phylogenetic analysis of SARS-CoV-2 revealed the similarity (75–88%) with bat SARS-like coronavirus.

**Conclusion:**

The sign and symptoms of novel severe acute respiratory syndrome coronavirus 2 are also discussed in this paper. The worldwide outbreak and prevention from severe acute respiratory syndrome coronavirus 2 are overviewed in the present article. The latest variant of coronavirus and the status of vaccines are also overviewed in the present article.

## Background

Ongoing pandemic outbreaks (COVID-19) and public health crises were intimidating across the globe with the appearance and spread of the 2019 novel coronavirus known as SARS-CoV-2. SARS-CoV-2 (severe acute respiratory syndrome coronavirus 2) quickly spread from its origin in the Wuhan City of Hubei Province of the Republic of China, a country in East Asia, to the rest of the world (Wang et al. 2020a, b). Still, with small amounts of asymptomatic transmission between people, it spreads throughout the globe (Mohan et al. 2021). Coronaviruses (CoVs) are single-stranded, large, enveloped RNA viruses. They are spherical pleomorphic particles with bulbous surface projections. The CoVs genome size is from 29.8 to 29.9 kb (Su et al. 2016; Zheng et al. 2020). Virus particles have an average diameter of 12 μm (120 nm), an average envelope diameter of 0.08 μm (~ 80 nm), and spike length is 0.02 μm (~ 20 nm). The electron micrograph shows dense shells of an envelope with distinct electron pairs (Fehr and Perlman 2015).

CoVs have been reported in both avian hosts and various mammals, including bats, camels, dogs, and masked palm civets. They are earlier regarded as pathogens that only cause mild diseases in people having weak natural immune systems until the occurrence of the coronavirus causing severe acute respiratory syndrome (SARS-CoV) in late 2002 (Zhong et al. 2003; Drosten et al. 2003; Fouchier et al. 2003; Khailany et al. 2020). At present, at least seven coronavirus species are known to cause diseases in humans. Four species of this virus, i.e., 229E, OC43, NL63, and HKU1, only cause common cold symptoms. Severe illness can be caused by the lasting three viruses, which resulted in the outbreak of SARS in 2002–2003, when a new coronavirus of the β genera and with source in bats crossed over to humans via the transitional host of palm civet cats in the Guangdong province of China (Zhong et al. 2003; Drosten et al. 2003; Singhal 2020). Coronaviruses were responsible for the Middle East respiratory syndrome (MERS-CoV), which emerged in 2012 and remains in circulation in camels (Zaki et al. 2012). SARS-CoV-2, the virus emerged in December 2019 in Wuhan of China. To date, a total of 413,503,790 cases of COVID-19 have been reported across the globe which lead to the death of 5,826,562 people. An overview of SARS-CoV-2 transmission, timeline and pathophysiological properties were described by Hembram (2021) and they also depicted the host and SARS-CoV-2 interaction and explained how the virus exploits host machinery. This article will summarize an overview of SARS-CoV-2, its outbreak history, its taxonomy, and genomic information of SARS-CoV-2 available to date. The infected patients' signs and symptoms, transmission mode and diagnosis, and the status of chemotherapeutic drugs and vaccines being used for the treatment of its infection of SARS-CoV-2 will also be highlighted in the review paper.

## Main text

**Fig. 1:**
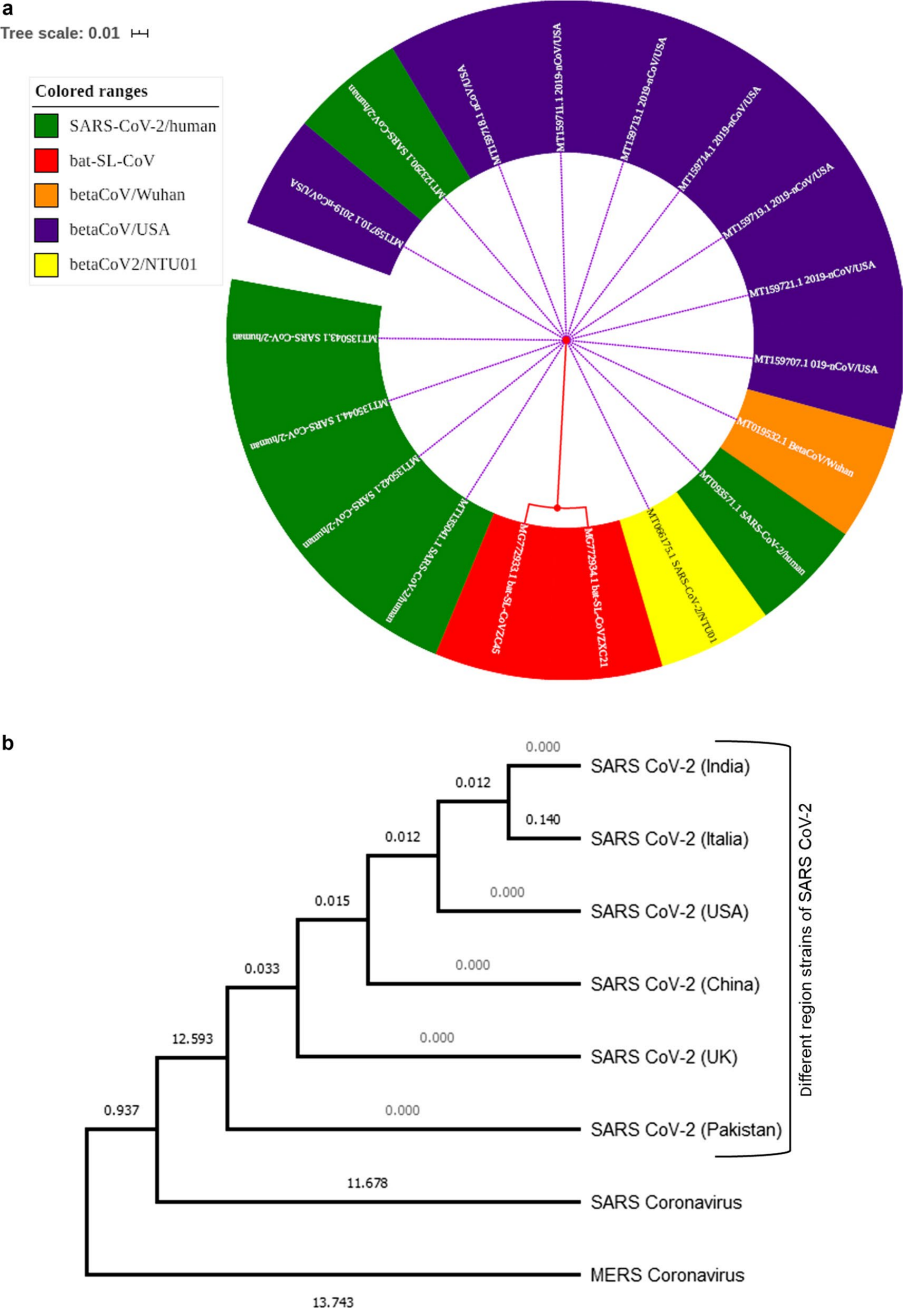
**a** Different coded colors show phylogenic relations among all organisms which were selected from the database, in which Bat-SL-CoVZC45 (Acc#MG772933.1) and Bat-SL-CoVZXC21 (Acc#MG772934.1) remarks as an origin to expend with nearest different neighbor stains of humans. **b** Phylogenetic tree of different regional strains of SARS-CoV-2 and related viruses. The numbers refer to the distance values to the reference sequence and the percentage of data coverage for internal nodes. Evolutionary analyses were conducted in MEGA software

The CoVs belong to the coronoviridae family within Nidovirales, and these viruses replicate in a set of nested mRNA. The subfamily of CoVs is divided into four general: alpha (α-CoVs), beta (β-CoVs, gamma (δ-CoVs), and delta (γ-CoVs), which includes the number of viruses in nature with their existence in birds and mammals (Fehr and Perlman 2015). Four genera of CoVs with their species is as follows: (1) Alphacoronavirus with species (Human coronavirus NL63, Miniopterus bat coronavirus, Miniopterus bat coronavirus HKU8, Porcine epidemic diarrhea virus, Human coronavirus 229E, Scotophilus bat coronavirus 512, Rhinolophus bat coronavirus HKU2) (2): β-CoVs with species ( β-CoVs1[OC43 HCoVs), Maurine coronavirus, Pipistrellus bat CoV HKU5, Rousettus bat coronavirus HKU9, Human coronavirus HKU1, Hedgehog coronavirus 1 (EriCoV), (SARS-CoV, SARS-CoV-2), Middle East respiratory syndrome-related coronavirus, Tylonycteris bat CoV HKU4, (3). gamma (δ-CoVs) with species: Beluga whale CoVs SW1, Infectious bronchitis virus. (4) Deltacoronavirus with species: Porcine coronavirus HKU15, Bulbul CoV HKU11 (Fan et al. 2019) (Fig. 1a, b).

### Novel coronavirus SARS-CoV-2 and its origin

Human coronaviruses were first shown to be linked to a high respiratory risk of infection in adults and children in the 1960s and display signs of cold or flu that have no substantial effects since their formation. Death and morbidity are alarming and worldwide aggravated by a severe coronavirus and the Middle East (MERS-CoV) acute respiratory syndrome in the twenty-first century (Paules et al. 2020). In a recent review, HCoVs had shown mild phenotypes in humans. They were modified when typical pneumonia in China's Guangdong province caused the virus to spread through overseas travel to dozens of other countries (de Wit et al. 2016). The disease was known as severe acute respiratory syndrome (SARS) caused by SARS-CoV (beta HCoV). Contacts of humans and animals at live games through virus zoonotic transmission were suspected (Song et al. 2019). In December 2019, however, China was recognized as highly pathogenic HCoVs (SARS-CoV-2). In Wuhan's capital Hubei and the central transport center of China, severe pneumonia of unknown origin caused by severe illness and death began to present to local hospitals. China informed the World Health Organization of the outbreak on December 31, 2019, and the Huanan Marketplace was closed on January 1. On January 7, the virus was described as a coronavirus with > 95% bat coronavirus homology and > 70% SARS-CoV similarity (Singhal 2020). From China, this virus started spreading to neighboring countries Thailand, Japan, and South Korea in quick succession. In the present situation, coronavirus 2 (SARS-CoV-2) has taken pandemic shape, and the USA is a highly affected country with this virus (total number of cases reported to date: 48,072,898).

A severe acute Coronavirus 2 (SARS-CoV-2) disease (COVID-19) of 2019 is associated with the Beta coronavirus family, which belongs to this group and shows the result of an extreme acute respiratory syndrome. At 125 nm in size, it is more significant than MERS, SARS, and influenza viruses. SARS-CoV-2 and SARS-CoV use the same receptors, angiotensin-converting enzyme ll (ACE2) (Zhou et al. 2020). Although natural reservoirs for human transmission remain unclear (Wei et al. 2020), some studies explained that spike protein in SARS-CoV-2 is identical to pangolin virus (Lam et al. 2020; Wong et al. 2020). The spike (S) surface glycoprotein for binding host cell receptors is critical for restricting host range (de Wilde et al. 2017).

### Structural information and Genome organization of SARS-Cov-2

Coronaviruses are considered the most extensive known RNA virus (Chen et al. 2020). Nowadays, the databases are flooded with whole-genome sequence data of SARS-CoV-2 and more than 1500 whole-genome sequences around the world are available with the NCBI database. It is mainly possible due to advanced next-generation sequencing technology. The genomic information of SARS-CoV-2 is encoded within single-stranded RNA (+ sense) of approximately 29.8–29.9 kb in size. Overall the genome consists of a 5’-cap, 5’-untranslated region (UTR), open reading frames, a 3’-UTR, and 3’-poly (A) tail. The genomic information is encoded by mainly three categories of proteins, viz. nonstructural proteins (NSP), structural proteins, and accessory proteins. The central portion of the genome which is over two-thirds, typically code for nonstructural proteins whereas only one-third code for primary structural proteins and accessory proteins. The detail of each gene is explained in Table 1 (based on Khailany et al. 2020 and Accession no-MT396242 of NCBI database).

**Table 1:** Major genes present in the genome of SARS-CoV-2 till data

S. no.	Size	Gene name	Protein type
1	265	5’ UTR	–
2	21,290	ORF1ab gene	ORF1ab polyprotein and ORF1a polyprotein (nonstructural protein)
3	3822	S gene	Surface glycoprotein (Structural protein)
4	828	ORF3a gene	ORF 3a protein (Accessory protein)
5	228	E gene	Envelop protein (Structural protein
6	669	M gene	Membrane glycoprotein (Structural protein
7	186	ORF6a gene	ORF6a protein (Accessory protein)
8	366	ORF7a gene	ORF7a protein (Accessory protein)
9	132	ORF7b gene	ORF7b protein(Accessory protein)
10	193	ORF8 gene	ORF8 protein(Accessory protein)
11	908	N gene	Nucleocapsid phosphoprotein (Structural protein)
12	117	ORF10 gene	ORF10 protein(Accessory protein)
13	229	3’UTR	–

**Fig. 2:**
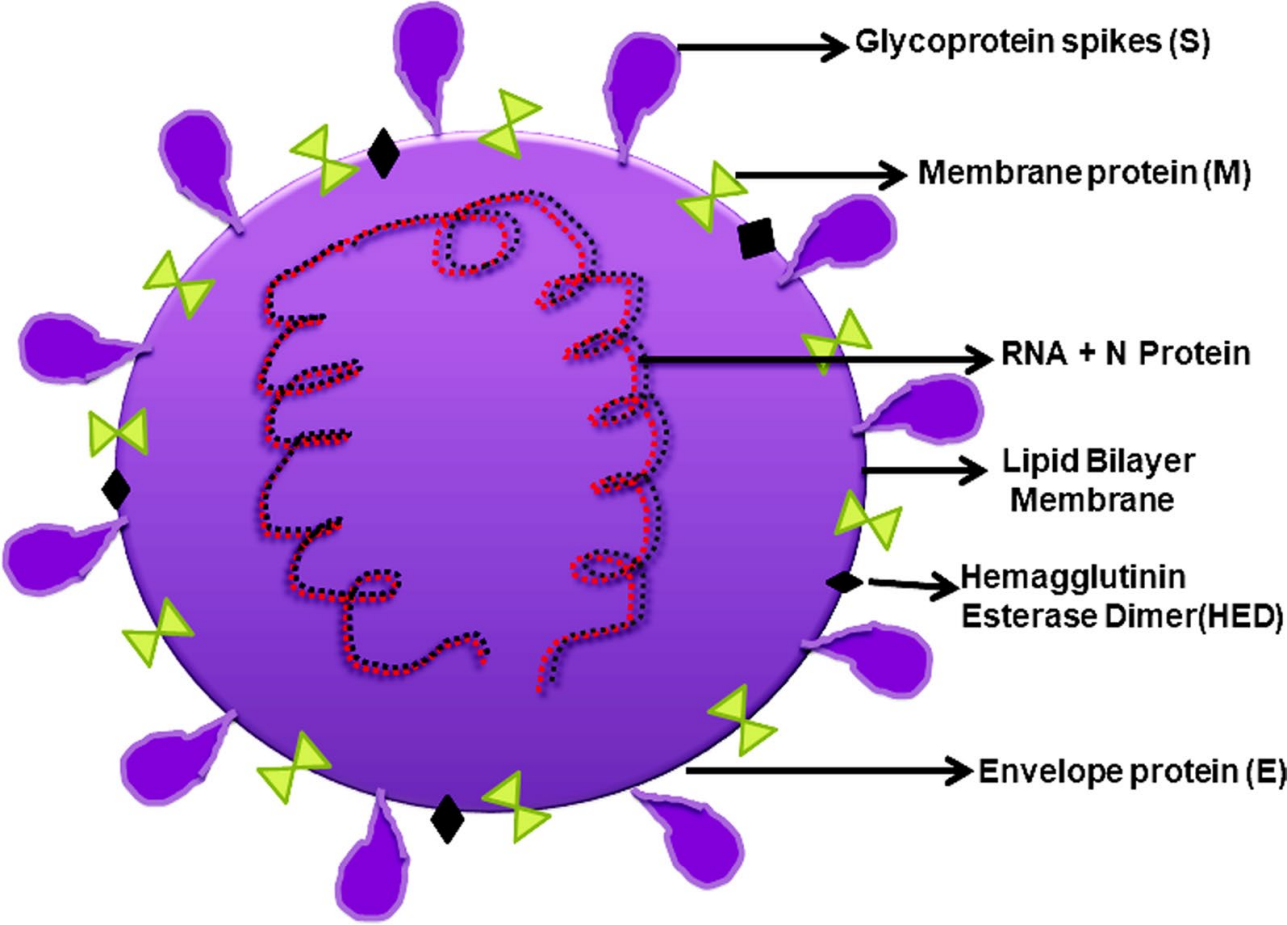
Structural information of SARS-CoV-2

The first gene from the 5’ side is the ORF1ab gene which codes for two overlapping ORF and translates into ORF1ab polyprotein and ORF1a polyprotein. It codes for various nonstructural proteins. ORF1ab gene is also designated as replicas/transcriptase. ORF1ab polyprotein includes a total of 15 peptides and covers the entire ORF1ab gene. The gene encoded are leader protein (nsp1), nsp2, nsp3, nsp4, nsp5 (3c-like proteinase, also known as cysteine proteinase), nsp6, nsp7, nsp8, nsp9, nsp10, nsp12 (RNA-dependent RNA polymerase), nsp13 (helicase), 3’-5’ exonuclease (nsp14). Endo RNAse (nsp15) and 2’-o-ribose methyltransferase. ORF1a polyprotein, another ORF of the ORF1ab gene, is encoded by an initial 13,218 nucleotide. ORF1a polyprotein includes peptides from leader protein to nsp10 and an additional nsp11 (https://www.ncbi.nlm.nih.gov/nuccore/1835438183). The rest one-third portion of the genome encodes primary structural proteins and accessory proteins. The structural proteins include highly conserved spike (S) protein, membrane (M) protein, envelope (E) protein, and nucleocapsid (N) protein (Fig. 2). The entry of the virus to the host cell is mediated through the binding of the S protein to the host receptor, whereas M, E, and N proteins are part of the nucleocapsid of viral particles. It also consists of six accessory proteins ORF3a, 6a, 7a, 7b, 8, and 10 genes (Li et al. 2005; Oostra et al. 2007).

In recent times a lot of research work is going on across the globe related to SARS-CoV-2. WHO is bringing the best scientists and health professionals together on a global level to boost the research and development process, and develop new norms and standards to contain the spread of the coronavirus pandemic. Khan et al. 2020, had done a comparative genome analysis of novel coronavirus (SARS-CoV-2) from different geographical locations and revealed genome sequences of SARS-CoV-2 displayed high identity (> 99%) to each other, while they shared 82% identity with SARS-CoV. Identification of RNA-dependent RNA polymerase (RdRp), also known as nsp12, is a core component of the virus replication with SARS-CoV-2 was done, and complete conformation of RdRp resembles the SARS-CoVRdRp (Subissi et al. 2014; Kirchdoerfer et al. 2019; Elfiky 2020). The molecular architecture of SARS-CoV-2 was seen by Yao et al. in 2020 and 18,500 RNP were found in the viral lumen and 56,832 spikes from the viral particles were physically identified, about 97 percent in the conformation of a prefusion and 3 percent in the conformation of the postfusion. SARS-CoV-2 was not associated with synonymous (K/K) transition. These were 1,008 or 1,094 and it has been located at nine special high linkages, and four significant haplotypes were found: H1, H2, H3 and H4 (Bai et al. 2020).

A recent analysis of the virus's origin showed an unexplained preference source for some recipients and clinical spectrums. A scientist from worldwide has been engaged in genomic isolation as approximately 16 full-length genome sequence projects were completed in 2019, ten in China and six in the USA, and several other countries worldwide. The codon test reveals that serpents are the natural host of these viruses. Still, the current study rejected the hypothesis of this publication and reported that it was caused due to Bat_ Severe Acute Respiratory Syndrome (SARS)-like CoVs within the subgenus Sarbecovirususing phylogenetic analysis relationship. Nidom et al. (2021) investigated the full-length genome mutation analysis of 166 Indonesian SARS-CoV-2 isolates before the vaccination program. They focused on unlocking the mutation of S protein in all the collected isolates. They described every single mutation in the S protein of all the isolates and found that out of 166 isolates, D614G mutation appeared in 103 Indonesian SARS-CoV-2 isolates. In this review, we also performed the phylogenic analysis using bioinformatics tools and techniques for evolutionary relationships of 2019-nCoV based on the availability of a complete genome in public resource databases. The sequence pairwise identity was calculated with online Clustal Omega facilitated by the European bioinformatics institute (https://www.ebi.ac.uk/) and aligned using ClustalW methodology; we use whole-genome strains of Bat SARS-like coronavirus (Accession no. MG772933.1) for phylogenetic analysis, which was matched with available 2019-nCoV, SARS-CoV-2 outbreak strains from different regions mentioned in Fig. 1 presented with iTol online tool (https://itol.embl.de/) and labeled name with other colored codes, among all, the red-colored show Bat-SL-CoVZC45 (Acc#MG772933.1) and Bat-SL-CoVZXC21 (Acc#MG772934.1) as an origin to expend with nearest different neighbor stains of humans available on NCBI database from China, USA, India, and Canada all ids and accession numbers are mentioned in the diagram. We also observed that both Bat_SARS_likeCoVs are highly similar to the above-mentioned stain as approximately 75 to 88% similar to the nucleotide sequence of 2019-nCoV and 2019-nCoV-2 from Wuhan (China) and the USA. Our findings reports were matched with the latest published research on Bat_SARS-like CoV (Zhou et al. 2020).

**Fig. 3:**
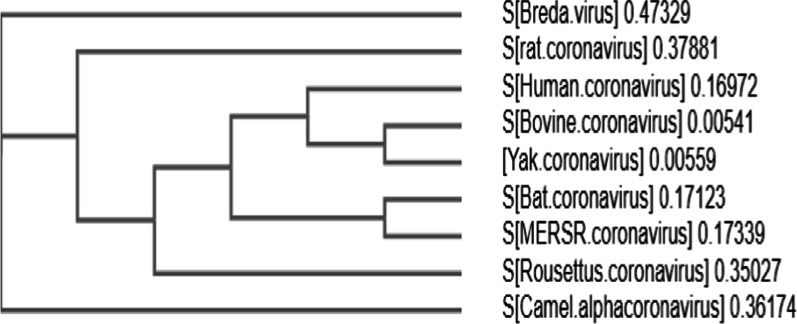
Spike glycoprotein protein-based phylogenetic analysis of SARS-CoV-2

We additionally explored the further phylogenetic analysis for targeting the Spike glycoprotein of CoVs from human Middle East respiratory syndrome-related coronavirus (ASU90549.1; AGW27881.1), Rousettus bat coronavirus(AOG30822.1), animal-origin CoVs comprising MERSV camel(ALA50193.1), Breda virus(AAS17959.1), Lucheng Rn rat coronavirus (APU57645.1), Bovine coronavirus (QBG67080.1) bat coronaviruses (YP_009361857.1), and the current outbreak nCoVs from different regions. The sequences of nCoV are available in the NCBI database till April 10 2020 were retrieved. The Bat_SARS-like CoVs show 100% bootstrap supporting values with 2019-nCoV quarantines of the current outbreaks (MERSR) with 0.17 identical value based on Clustal W alignments, the sequence identity of 2019-nCoV strains exposed Bat SARS-like CoVs as the nearest neighbors sequence identity on the amino acid basis mentioned in Fig. 3. Overall, the nucleotide and amino acid-based percent identities indicate toward highly diverged nature of novel coronaviruses.

**Fig. 4:**
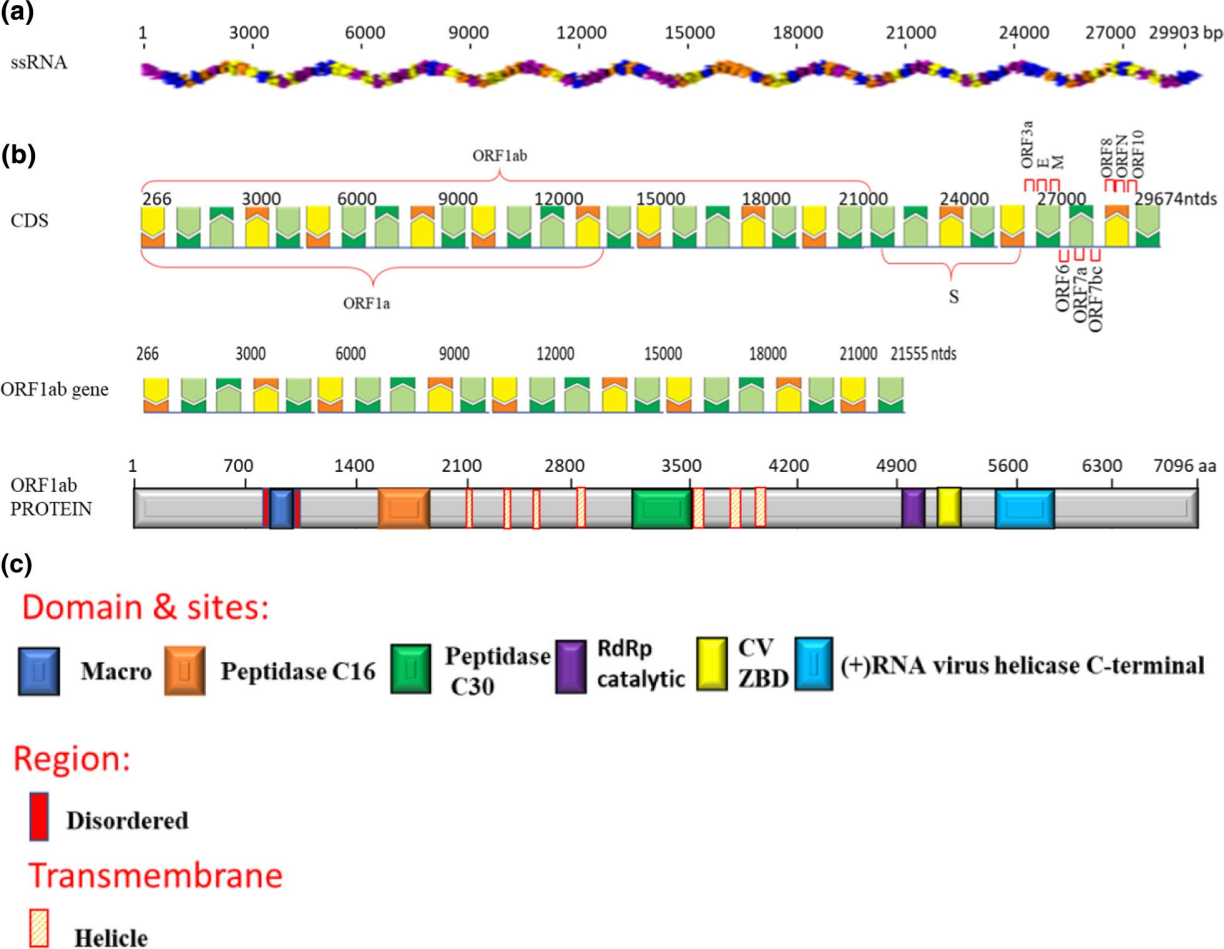
**a** Genomic RNA of SARS-CoV-2 isolates Wuhan-1 (NC_045512). **b** Coding sequences of SARS-CoV-2. **c** ORF1ab polyprotein with Regions, Transmembrane and Domain sites of Macro, Peptidase C16, Peptidase C30, RdRp catalytic, CV ZBD and (+) RNA virus helicase C-terminal

**Fig. 5:**
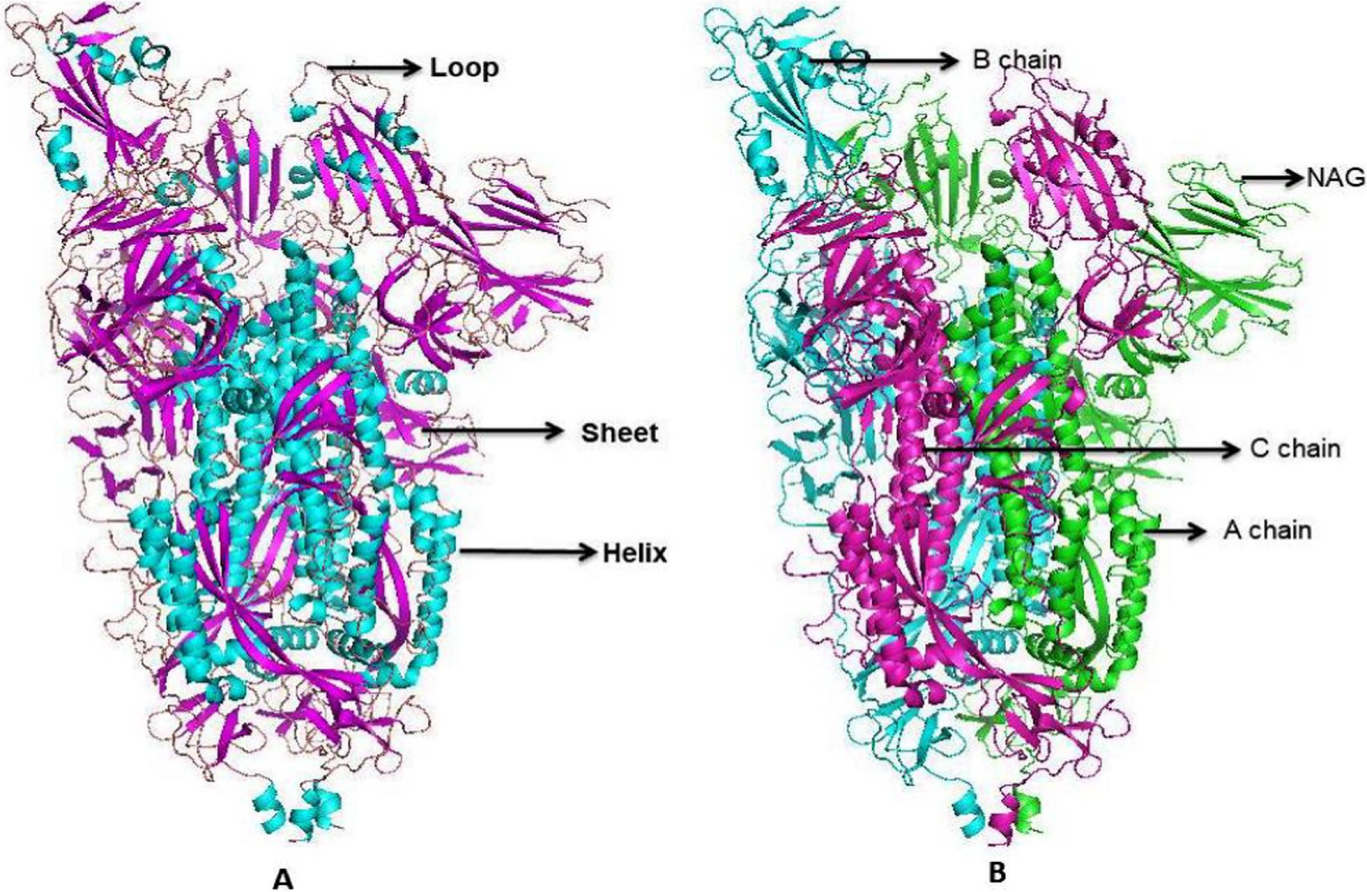
A tertiary structure of spike (S) protein and Membrane (M) protein of SARS-CoV-2

Diverse aspects of transcription and replication are involved (Gorbalenya and Koonin 1993). The detailed insight of ssRNA (Genomic RNA of SARS-CoV-2 isolate Wuhan-1 (NC_045512).), CDS (Coding sequences of SARS-CoV-2 (GeneID: 43,740,578) (Wu, Zhao et al. 2020)), and protein (ORF1ab polyprotein (YP_009724389.1) with Regions, Transmembrane and Domain sites of Macro (1003–1169), Peptidase C16 (1611–1875), Peptidase C30 (3241–3546), RdRp catalytic (4981–5143), CV ZBD (5302–5385) and ( +) RNA virus helicase C-terminal (5558–5909) shown in Fig. 4 as describe in Uniport server (https://www.uniprot.org/). The tertiary structure of spike (S) protein and membrane (M) protein are given in Fig. 5. A prediction of surface protein regions (antigenic epitopes sites) for spike (S) protein and membrane (M) protein was made using an online tool (http://sysbio.unl.edu/SVMTriP/index.php) (Table 2) that are favorably recognized by antibodies (antigenic epitopes) can help the design of vaccine components and immuno-diagnostic reagents (Yao et al. 2020a). Lan et al. (2022) reported the secondary structure heterogeneity of the entire SARS-CoV-2 genome in two lines of infected cells at single-nucleotide resolution and revealed the alternative RNA conformations across the genome and at the critical frameshifting stimulation element (FSE) which were drastically different from prevailing population average models. Their work may form the basis of coronavirus RNA biology and help in designing the SARS-CoV-2 RNA-based therapeutics.

**Table 2:**
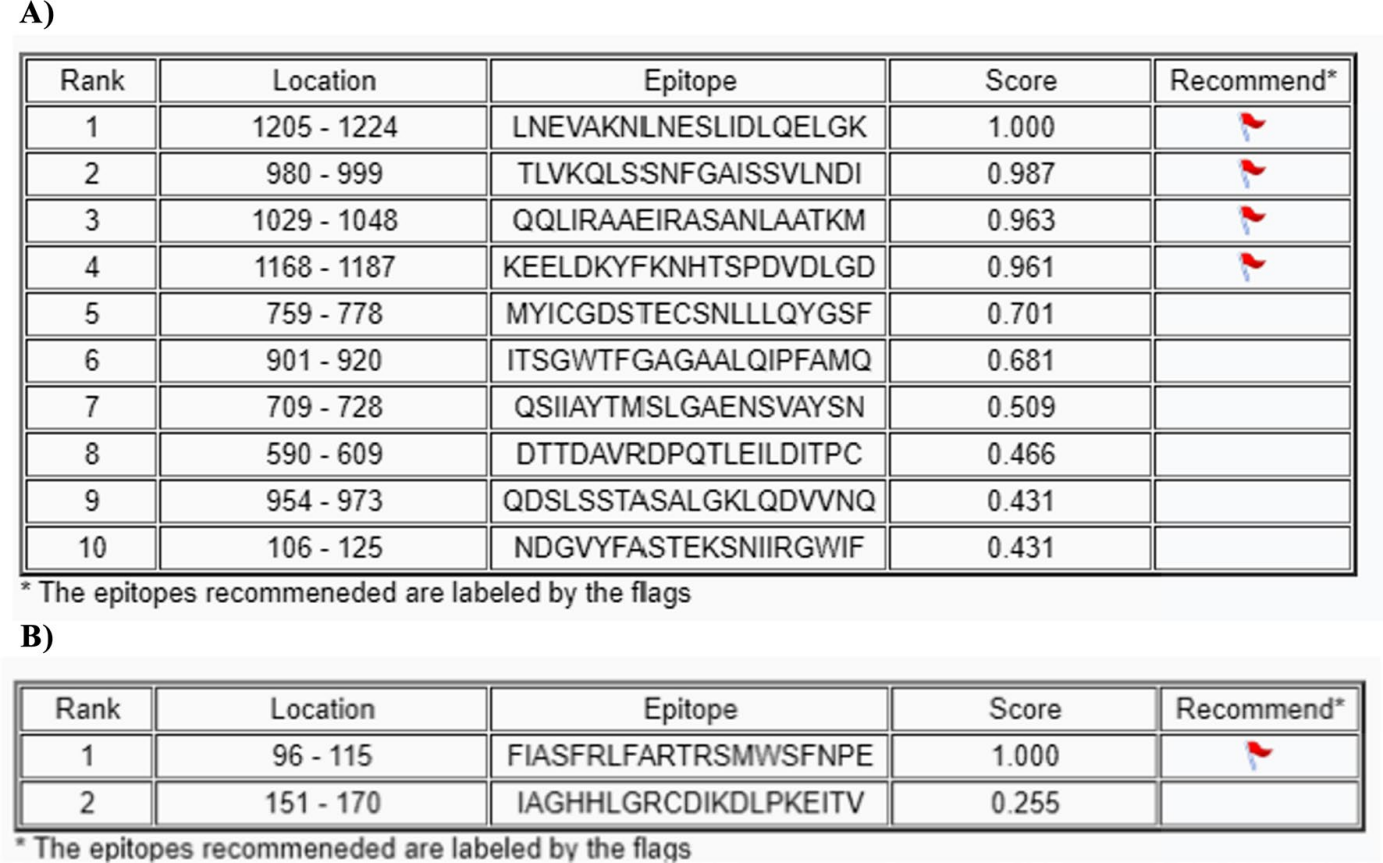
Prediction of a ntigenic epitopes sites present in spike (S) protein (A) and M protein (B) surface of SARS-CoV-2A)

### Life cycle of coronavirus

The receptor recognition and fusion of membranes are two crucial functions of spike proteins (S) in all coronaviruses (Li 2016). S glycoprotein attaches to the receptor of host cells to initiate infection; after this, cleavage and activation of attached S are triggered by protease. Depending on protease activation and division, there will be endocytosis for the virus entry into a cell or viral envelope, and the host membrane can be directly fused. Once a virus particle enters the cell, it is uncoated, followed by genome entry into the cytoplasm. The RNA genome of coronavirus has a 3′ adenylated tail and 5′ methylated cap, allowing RNA with the ribosome of the host cell for further undergoing translation. Translation of virus genome with open reading frame forms polyprotein. The polyprotein is then cleaved by its protease into multiple proteins, which are nonstructural (Fehr and Perlman 2015). Replication of the viral genome is critical in the complex of coronavirus. RNA polymerase, which is RNA-dependent, is involved in the synthesis of genomic RNA. Transcribing the viral genome is another significant function of the complex. An enzyme is further engaged in the synthesis of sub-genomic RNA; then positive strand behaves as the virus's progeny. After overlapping of the reading frame, mRNAs are the gene transcripts. Host ribosomes are then used for accessory and structural protein synthesis. The endoplasmic reticulum is used for translation, and S, E, and M (structural proteins) show direct protein interactions for virus assembly (Fehr and Perlman 2015).

### Novel SARS-CoV-2-related diseases and its sign and symptoms

COVID-19 symptoms include nasal congestion, dry cough, fever, fatigue, sore throat, and diarrhea. The most common clinical sign and symptoms were cough (75%), fever (91.7%), gastrointestinal symptoms (39.6%), and fatigue (75.0%), whereas the most common comorbidities were diabetes (12%), hypertension (30%). Several patients self-reported urticarial (1.4%) drug hypersensitivity (11.4%), and none of the patients reported allergic diseases or asthma. Chronic obstructive pulmonary disease (COPD, 1.5%) patients and smokers (1.5%) were rarely seen. Radiological findings showed the most common sign of patchy opacity or (89.6%) of bilateral ground glass (Zhang et al. 2020).

Data were analyzed from January 21 to February 14 from 65 deceased patients and 96 survivors (COVID-19 confirmed). The clinical presentation showed that the main coexisting health issues were endocrine system (22 [43%]) and cardiovascular (36 [60%]) diseases. At onset of illness most common symptoms were Fever (59 [91%]), anorexia (54 [86%]) short of breath (65 [100%]), cough (42 [65%]) fatigue 56 [86%]), 80% of the deceased patients were old (60 years), and 65% were male. Comorbidities among 65 deceased patients were acute heart injury (3 [5%]), acute respiratory distress syndrome (ARDS) (27 [42%]), acute respiratory injury (31 [48%]), septic shock (1 [2%]), and acute renal injury (2 [3%]) compared to (1%) surviving patients with nonsignificant ARDS (Li et al. 2020a; b, c). SARS-CoV-2 18 patients from Singapore showed clinical presentations with mild respiratory tract infection, some required oxygen supply, and antiretroviral treatment showed variable clinical outcomes. Some patients developed vomiting, nausea, and diarrhea with treatment (Young et al. 2020).

### Medical diagnosis techniques for early recognition of COVID-19

Medical researchers use MERS-CoV and SARS-CoV prototypes in diagnostic modalities for the early recognition of COVID-19 cases and adopted several approaches to identify and diagnose the coronavirus in the suspected patients. The methodology is given below.

RT-PCR: quantitative reverse transcription PCR (RT-qPCR) is used when the starting material is RNA, and the genetic material of coronavirus is RNA. MylabPatho Detect COVID-19 Qualitative PCR kit is the first Made in India testing kit to get approved by the Central Drugs Standard Control Organization (CDSCO) and Indian Council of Medical Research (ICMR). The experimental analysis revealed that the Ct values for positive SARS-CoV-2 samples should be no higher than 37.

PCR-PoC or Rapid PCR test: Cepheid has designed an automated molecular test for the qualitative detection of SARS-CoV-2. They leveraged the design principle of cartridge technology, in which multiple regions of the viral genome are targeted. The kit (Xpert Express SARS-CoV-2 test kit) provides rapid detection in as soon as 30 min for positive results and less than a minute to prepare the samples.

PCR-Isothermal Amplification: A TILA BioSystems has developed the iAMP Covid-19 detection kit based on isothermal amplification technology (temp 60–65 °C), which can detect viral RNA directly from samples without prior RNA extraction process. They designed the primer sets to detect RNA and later cDNA from the N and ORF-1ab genes of the SARS-CoV-2 virus in nasal and oral swabs.

PCR-LAMP: Single tube technique for the amplification of DNA using 4–6 primers which form loop structures to facilitate subsequent rounds of amplification. IN INDIA, current PCR kits enable detecting the E gene for screening and the RdRp (RNA-dependent RNA polymerase) gene for confirmation. SreeChitraTirunal Institute for Medical Sciences and Technology, Trivandrum, has developed a diagnostic test kit “ChitraGeneLAMP-N” which detects the N Gene of SARS-CoV-2 and allows confirmation in one test without the need for a screening test.

Antigen-Based Kits: The antigen-based kits look for the proteins on the surface of the virus. The paper strips of these kits contain artificial Abs that bind to the antigens. SD Biosensor has developed “*Standard F COVID-19 Ag FIA*,” a fluorescent immunoassay to detect SARS-CoV-2 infection in human nasopharyngeal swab specimens, identifying the existence of SARS-CoV-2 viral nucleoprotein antigens, and this kit can provide fast results as soon as 30 min.

Antibody IgG/IgM detection Kits: BioMedomics has launched the “*BioMedomics Rapid IgM-IgG Combined Antibody Test*” for COVID-19 to qualitatively detect IgG and IgM antibodies of the novel coronavirus in human serum, plasma, or whole blood in vitro. This technique is considered the world’s first rapid point-of-care lateral flow immunoassay to aid in diagnosing coronavirus infection.

Next-Generation Sequencing: YOUSEQ has developed a complete kit for amplicon-based NGS Library preparation for the novel COVID19 virus. This kit is ideal for monitoring viral populations and mutation events that target amplicons. This NGS kit is based on amplicon sequencing, making for a fast, easy protocol and exceptional sensitivity when working with low viral loads.

Animal models against 2019-nCoV are under process (Sheahan et al. 2020). However, different case studies (explained below) have been reported, demonstrating the novel virus diagnosis; Polymerase chain reaction (PCR) was used to confirm the SARS-CoV-2 in 18 patients from 4 hospitals in Singapore. Experimental data were collected using threshold cycles of PCR from viral shedding in nasopharyngeal swabs, stool, blood, and urine (Young et al. 2020). A total of 140 confirmed SARS-CoV-2 patients’ analysis showed eosinopenia (52.9%) and lymphopenia (75.4%) in most cases. Counting blood lymphocytes correlate with counts of eosinophil in non-severe patients. Severe patients were associated with significantly high levels of procalcitonin, C-reactive protein (CRP), and Di-dimer (Zhang et al. 2020).

Laboratory diagnosis of 65 deceased patients and 96 survivors (confirmed COVID-19) analyzed, 31% had leukopenia from 96 surviving patients compared to 8% in deceased patients. In symptomatic patients, the average lymphocyte percentage in the surviving group was lower than in the deceased patients. The average levels of creatinine, CK, urea, and nitrogen were higher in the deceased patients than in survivors. A significant difference was seen with CRP between survivors and the dead group Bronchitis, bilateral pneumonia, Bronchitis with ground glass opacity lesions multiple mottling was seen with CT images, in the lung of the deceased patient than survivors (Li et al. 2020a; b, c).

### COVID-19 drugs and vaccine status

Various countries struggled to develop a potential vaccine against novel coronavirus, as time was needed to access health professionals to minimize the health and life risks of patients. Now that COVID-19 vaccines have reached billions of people worldwide. Meanwhile, alternate treatments were used to cope with this disease (Corman et al. 2020). According to recommendations of the world health organization, the available drugs that could be a possible treatment for COVID-19 patients include lopinavir, remdesivir, ritonavir, nitazoxanide. The patient must obtain fluid therapy, oxygen therapy, and various antibiotics, such as clarithromycin, azithromycin, and erythromycin, to treat associated bacterial infections. Moreover, if given right after the symptoms appeared, antiviral drugs can decrease the spread of disease to other persons by minimizing the shedding of the virus through the patient’s respiratory secretions. Moreover, the target-specific treatments of contacts also reduce the hazard of being infected (Welliveret al. 2001).

Many general approaches might be used to identify a possible antiviral therapy for the coronavirus, a human pathogen. The first is to evaluate current wide-spectrum antiviral medicines using standardized tests used to treat certain viral infections. The second approach includes scanning a chemical library including several known molecules or libraries, including various cell lines. The third method provides for the synthesis of new particular medicines focused on human coronaviruses' biophysical and genome knowledge (Channappanavar et al. 2017). Furthermore, the execution of prophylaxis and antiviral therapies has various necessities. The availability of drugs must be sufficient; it must be cost-effective and maintain maximum application safety (Agostini et al. 2018).

Due to its receptor recognition and membrane fusion role, the SARS-CoV S protein is considered the primary target to develop a vaccine against SARS-CoV infection. S protein induces immunization by activating T-cell immune responses and neutralizing antibodies that block viral binding and fusion into the membrane, thereby neutralizing infection of the virus (Park et al*.* 2020). Various S protein-centered approaches such as the practice of full length or suitable part of S protein, recombinant S protein-containing receptor-binding domain (RBD), and recombinant vectors encoding RBD had been adopted to produce coronavirus vaccines (Belouzard et al. 2012).

Neuraminidase inhibitors such as oseltamivir, peramivir, and rapivab can block viral neuraminidase protein and prevent the viral release and infection of new host cells used as oral drugs in China to treat COVID-19 without concerning its efficacy (Wang et al. 2020a, b). Neuraminidase inhibitors could not be utilized against novel coronavirus as a coronavirus. However, various antiviral drugs such as nitazoxanide, ribavirin, peniclovir, chloroquinone, remdesivir, nafamostat, and favipiravir have been tested in vitro for their antiviral efficacy to control COVID-19 infection. The drugs known as Chloroquine, remdesivir, favipiravir, and arbidol have been under clinical trials, and encouraging results concerning their safety and efficacy have been attained hitherto (Dong et al. 2020).

RNA synthesis inhibitors, Nucleoside analogs, peptide (EK1), broad-spectrum antiviral drugs (remdesivir and favipiravir), and anti-inflammatory drugs could be utilized as the possible alternatives to treat nCOV-2019 (Lu 2019). Convalescent plasma treatment had considerably limited patients' death and viral load during SARS-CoV infection and is hence utilized in china to treat SARS-CoV-2 disease. Even though outcomes were encouraging, safety and efficacy considerations needed to be scrutinized (Zhang and Lu 2020).

Drug repositioning (also known as drug repurposing) strategy that utilizes commercially available and approved medicines such as antiviral drugs, drugs to treat autoimmune disorders, and the utilization of standardized antibiotics has been applied to 2019-nCoV patients (Beck et al. 2020). Reprocessing medicines has multiple advantages, including cost-effectiveness, safety responses, broad accessibility, and health professionals' experience in existing medications; (Lee et al. 2003). Antimalarial and HIV treatment drug chloroquine lopinavir/ritonavir were tested in this regard to their efficacy and protection choices against patients with new coronavirus (Covid-19); the clinical studies were commonly used for practice to exhibit anti-coronavirus response against SARS-CoV patients (Huang et al. 2020).

An authorized hydroquinone antimalarial drug against chemoprophylaxis has been widely used to treat malaria and other autoimmune-related diseases (Agostini et al. 2018). It also expresses antiviral potential against novel coronavirus (Yao et al*.* 2020a, b). Murabutide is considered a remarkable immune-stimulating drug that could be proved as a potential drug against coronavirus (Galvez et al. 2020). Bacillus CalmetteGuérin (BCG) immunization had been shown effective response against respiratory problems by providing extensive results during sepsis and various viral infections. It had been reported that countries with BCG immunization policies are less affected as compared to countries without BCG vaccination policies (Italy, USA, and Netherland) which suggests the BCG vaccination possible treatment against COVID-19 (Miller et al. 2020). Teicoplanin is an antibiotic for bacterial disease, which has shown potential effectiveness against the use of SARS-CoV-2 as a possible alternative for the treatment of the 2019-nCOV antibiotic and of some viruses, including influenza, Ebola virus, HCV (hepatitis C virus), HIV, flavivirus, and coronavirus, like SARS-CoV and MERS-CoV (Baron et al. 2020). However, safety and efficacy considerations need to be evaluated.

Some of the vaccine maker biotech companies that initiated the vaccine production are discussed below.

The US-based Moderna completed manufacturing of clinical trial material for its variant-specific vaccine candidate, mRNA-1273.351, against the SARS-CoV-2 variant known as B.1.351 in first quarter of 2021 and shipped doses to the NIH for a Phase 1 clinical trial that will be led and funded by NIAID. Takeda Pharmaceutical Co., Ltd submitted a New Drug Application to the Government of Japan’s Ministry of Health, Labour and Welfare (MHLW) to import and distribute Moderna’s vaccine candidate against COVID-19 in Japan.

One another Australia-based NovavaxInc has introduced a vaccine candidate, NVX-CoV-2 373. The vaccine candidate has been engineered from a genetic sequence of the SARS-CoV-2 virus, and single and double doses of the vaccine showed great promise in baboons and mice. On January 28, 2021, NVX‑CoV2373 became the first protein-based vaccine candidate to demonstrate clinical efficacy against the original strain of COVID-19 and both of the rapidly emerging variants in the UK and South Africa. In addition, the Company announced the final efficacy of 96.4% against mild, moderate, and severe disease caused by the original COVID-19 strain in a pivotal Phase 3 trial in the UK. Novavax COVID-19 vaccine brands include Nuvaxovid, Covovax, NVX-CoV2373. The Covovax version of the Novavax vaccine was authorized in Indonesia on November 1, 2021.

INOVIO Pharmaceuticals developed an INO 4800 DNA-based vaccination product at the San Diego Laboratory and concluded phase 1 of the 40 healthy people using the clinical trial. For depth understanding in phase 2, research on 40 voluntaries was scheduled for a shorter period. In November 2021, INOVIO received US FDA authorization to conduct phase 3 trial in the US INOVIO is partnering with Advaccine Biopharmaceuticals Suzhou Co., Ltd. to conduct the phase 3 segment in multiple countries in the Americas, Asia, and Africa.

Pfizer (US) has collaborated with a German company, BioNTech, to develop an RNA vaccine candidate for COVID-19. The vaccine was composed of nucleoside-modified mRNA (modRNA) encoding a mutated form of the full-length spike protein of SARS-CoV-2, which is encapsulated in lipid nanoparticles and supplied under the brand name “Comirnaty.” They started the clinical trials I to III in Germany from April 2020 to November 2020. In December 2020, the UK authorized its use on an emergency basis. Till September 2021, more than 1.5 billion COVID-19 vaccine doses have been shipped by the company worldwide.

Another major player in developing a potential vaccine is a China-based vaccine maker CanSino Biologics Inc. which has also manufactured an approved vaccine for Ebola. They are currently working on Adenovirus Type 5 Vector using a Non-Replicating Viral Vector platform, and till February 2021, all 3 clinical phase trials were conducted for this covid-19 vaccine (Ad5-nCoV brand name Convidecia). It is approved for use by some countries in Asia, Europe and Latin America.

Beijing-based Sinovac Biotech has also developed the covid-19 vaccine and already commenced a phase 1 trial. They tested their vaccine with two different doses on monkeys. Sinovac had previously developed a vaccine against SARS but had to stop the production at phase 1 as the disease was contained.

Another potential coronavirus vaccine, “ChAdOx1 nCoV-19,” is being developed by the Oxford University, UK in collaboration with British–Swedish company AstraZeneca. They injected the immunization to the monkeys infected with the SARS-CoV-2 virus and appeared to prevent damage to the lungs. The Oxford–AstraZeneca COVID-19 vaccine, code named AZD1222 and sold under the brand names Covishield and Vaxzevria. The clinical trial on humans already began in late April 2020 and on 30^th^ December 2020, the vaccine was first approved for use in the UK vaccination program. The first vaccination outside of a trial was administered on 4^th^ January 2021. Till March 2021 the vaccine was produced at several sites worldwide including the Serum Institute of India at Pune. By November 2021 more than 2 billion doses of the vaccine have been released to more than 170 countries worldwide.

An Indian company Bharat Biotech developed a Covid-19 vaccine in collaboration with the Indian Council of Medical Research (ICMR) and the National Institute of Virology (NIV). The vaccine received DCGI approval for Phase I and II Human Clinical Trials in July 2020. The vaccine is developed using Whole-Virion Inactivated Vero Cell derived platform technology and sold under the brand name COVAXIN which was India's first Indigenous COVID-19 Vaccine.

Gamaleya Research Institute of Epidemiology and Microbiology in Russia developed an adenovirus viral vector vaccine which was the world's first combination vector vaccine for the prevention of COVID-19 and was registered in August 2020 by the Russian Ministry of Health. The vaccine is sold under the brand name “Sputnik V.” Recently in December 2021, the developer stated that a booster shot of the Sputnik vaccine provides a stronger antibody response against the Omicron variant of COVID-19. Till August 2021, 1 billion doses of vaccine have been supplied across the globe.

### Vaccination statistics

According to the Press Information Bureau, Government of India, a total of 173.42 cr vaccine doses have been administered so far under Nationwide Vaccination Drive (February, 15, 2022, 9:10 AM by PIB Delhi). Out of total vaccine doses, 75.3 cr people have been fully vaccinated which confers the vaccination rate of 54.6%. The booster doses were also given to almost 16 cr people across the nation. A total of 1.04 TCr vaccine doses have been administered so far under Worldwide Vaccination Drive out of which 425 cr people are fully vaccinated.

### Coronavirus cases

**Fig. 6:**
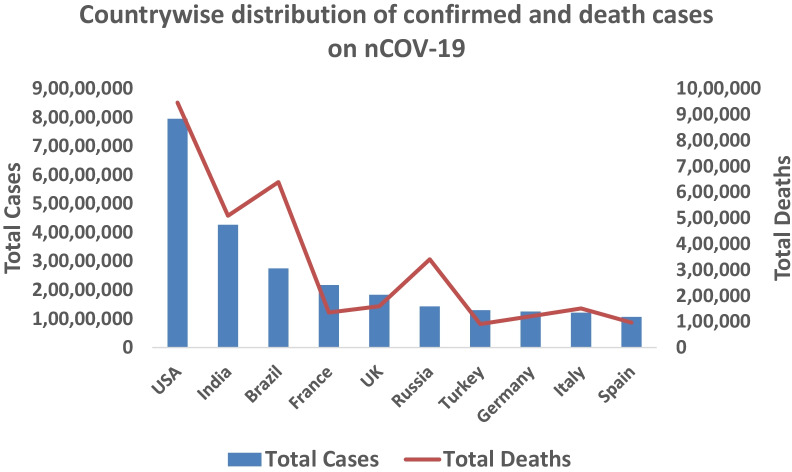
Number of confirmed and death cases of countries most affected by COVID-19 as of February 15, 2022

The outbreak of pneumonia-like infection has emerged in Wuhan city, Hubei province of China, in December 2019, later confirmed as a new form of SARS-CoV-2. WHO declared this disease as COVID-19—Coronavirus Disease-2019 (Yang et al. 2020). According to the WHO situation report on December 21, 2021, 275,893,455 cases of nCOV-19 have been reported in 213 countries worldwide which increased almost 6 times in one year period. The value has increased to 413,503,790 in just two months period (February 15, 2022). The total number of deaths reported across the globe was 5,379,651 which is 4.5 × compared to the previous year (December 2020) and the current status depicts a total death of 5,826,562. A year-wise comparison of total confirmed cases of nCOV-19 is done in this section. The most affected countries are the USA (confirmed cases 9,567,543 till November 2020 and 79,520,665 till February 2022; reported deaths 236,997 till November 2020 and 946,180 till February 2022), India (confirmed cases 8,267,623 till November 2020 and 42,692,943 till February 2022; reported deaths 123,139 till November 2020 and 509,388 till February 2022), Brazil (confirmed cases 5,554,206 till November 2020 and 27,541,131 till February 2022; reported deaths 160,272 till November 2020 and 638,913 till February 2022) (Fig. 6).

### Preventions of SARS-CoV-2

In the current scenario, COVID-19 risk factors are unknown; however, in some contexts, scientists believe that coronavirus was transmitted in humans via contaminated live animals like snakes, bats, and camel (Balboni et al. 2012; Memish et al. 2014; Ji et al. 2020). It has been reported that CoVs can be insignificantly provoked by IFNr (Recombinant) with the assistance of ribavirin (Falzarano et al. 2013). For the last decades, SARS and MERS persist the major infectious diseases (Wong et al. 2015), which were the root of public health problems and increase mortality rate ten times more than average worldwide (Lescure et al. 2020). Current surveys have shown that the rate of death of people with inadequate immune systems could rise by those with no symptoms even at the early stage (Cascella et al. 2022), that is why Immunocompromised induvial social distancing and advised avoiding public gatherings is the first and foremost way to stop the outbreak of this virus (Adhikari et al. 2020). The second leading way to prevent COVID-19 is to wash hands ideally with the assistance of hand sanitizers, cleansers (soap and water), or alcoholic mixtures (Chaudhary et al. 2020; Seymour et al. 2020). In the same contrast, the hands are touching tools all over the time, so avoid shaking with others (Sajed and Amgain 2020) and exercise to prevent the immediate effects of respirational complications (Kim and Su 2020). The face must be covered with the bowed elbow or with wipes during sneezing or coughing. The practices of masks (FFP3 masks, N95 masks) and protection of eyes, wearing gloves and gowns are mandatory for the person. Avoid a person who suffers from flu, fever, trouble breathing, or other indications recommended by the World Health Organization (WHO 2020).

### Omicron epidemiology

Many variant strains of nCOV-19 have emerged over the past year which includes Alpha (B.1.1.7), Beta (B.1.351), Gamma or P.1 (B.1.1.28) and Delta (B.1.617.2). Among them, varying numbers of substitutions have been reported in the N-terminal domain (NTD) and the receptor-binding domain (RBD) of the spike protein. Lim et al. (2022) developed an installation-free cloud workflow for robust mutation profiling of SARS-CoV-2 variants from multiple Illumina sequencing data. They reported that their workflow could automatically identify mutated sites of the variants along with reliable annotation of the protein-coding genes in a cost-effective and timely manner. On 26 November 2021, WHO designated variant B.1.1.529 a variant of concern, named Omicron, on the advice of WHO’s Technical Advisory Group on Virus Evolution (TAG-VE). This decision was based on the evidence presented to the TAG-VE that Omicron has several mutations that may have an impact on how it behaves, for example, on how easily it spreads or the severity of illness it causes. The Omicron variant has a larger number of mutations (> 30 substitutions, deletions or insertions) in the spike protein which made it escape from protection conferred by vaccines and therapeutic mAbs (VanBlargan et al. 2022). Till January 17, 2022, the total cases of Omicron reported in India were 8209, out of which 5100 were active and the remaining 3109 were reported as recovered cases (https://pib.gov.in). As of February 14, 2022, the UK had reported the highest number of SARS-CoV-2 Omicron variant cases, while in India total of 11,348 cases of omicron were recorded.

## Conclusions

SARS-CoV-2 spread easily across the globe through direct contact with infected people. COV-2 is a single-stranded RNA virus and its detailed structural information is presented in the manuscript. Nowadays, information is available about this epidemic disease COVID-19. This SARS-CoV-2 has become a significant challenge even for powerful countries and for their scientist. The whole world’s scientific and medical intelligence agencies are still working to find a cure for this disease like advanced vaccination. Further, if this virus transmission stops in the future, it will probably remain there in different forms, so research should not be stopped and cope with its upcoming new varieties. For this purpose, we should have complete awareness about the infection and prevention of these viruses. The purpose of this review article is to enhance the structural and genomic understanding of this virus. The various information such as signs and symptoms, transmission mode, diagnosis, drugs, and vaccination, provided in the present article may facilitate the researchers and organizations to move ahead in developing the best measures to treat it.

## Data Availability

Data sharing is not applicable to this article as no datasets were generated or analyzed during the current study.

## Abbreviations

CoVs: Coronaviruses
nCoV: Novel Coronavirus
SARS-CoV-2: Severe acute respiratory syndrome coronavirus 2
S: Spike protein
M: Membrane protein
HCoV: Human Coronavirus
MERS-CoV: The Middle East respiratory syndrome coronavirus
NCBI: National Center for Biotechnology Information
(+) RNA: Positive-sense single-stranded RNA virus
ORF: Open reading frame
ACE2: Angiotensin-converting enzyme ll
